# Interaction of microglia with infiltrating immune cells in the different phases of stroke

**DOI:** 10.1111/bpa.12911

**Published:** 2020-11-02

**Authors:** Daniel Berchtold, Josef Priller, Christian Meisel, Andreas Meisel

**Affiliations:** ^1^ Department of Experimental Neurology Charité – Universitätsmedizin Berlin Berlin Germany; ^2^ Department of Neuropsychiatry and DZNE Charité – Universitätsmedizin Berlin Berlin Germany; ^3^ UK DRI University of Edinburgh Edinburgh UK; ^4^ Institute for Medical Immunology Charité – Universitätsmedizin Berlin Berlin Germany; ^5^ Center for Stroke Research Berlin Charité – Universitätsmedizin Berlin Berlin Germany; ^6^ Neurocure Cluster of Excellence Charité – Universitätsmedizin Berlin Berlin Germany; ^7^ Department of Neurology Charité – Universitätsmedizin Berlin Berlin Germany

**Keywords:** lymphocytes, myeloid cells, neuroinflammation, stroke

## Abstract

Stroke, in association with its complications, is one of the leading causes of mortality and morbidity worldwide. Cerebral ischemia triggers an inflammatory response in the brain that is controlled by the activation of resident microglia as well as the infiltration of peripheral myeloid and lymphoid cells into the brain parenchyma. This inflammation has been shown to have both beneficial and detrimental effects on stroke outcome. The focus of this review lies on the functions of myeloid cells and their interaction with infiltrating lymphocytes in different phases of stroke. A detailed and time‐specific understanding of the contribution of different immune cell subsets during the course of cerebral ischemia is crucial to specifically promote beneficial and inhibit detrimental effects of inflammation on stroke outcome.

## Graphical abstract

Stroke is one of the leading causes of mortality and morbidity worldwide. Brain inflammation, mediated by myeloid and lymphoid immune cells, is an inherent component of stroke pathology and contributes to the functional outcome after ischemia.

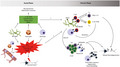

## Introduction

Stroke is one of the leading causes of death worldwide. In 2016 alone, approximately 5.6 million people worldwide died of stroke. Furthermore, the incidence of stroke is steadily increasing and prognoses predict 12 million deaths in 2030 (36, 117). One of the main complications following stroke are stroke‐associated infections, notably pneumonia. Stroke‐associated infections occur in approximately 30% of patients and pneumonia in up to 10% of stroke cases (15, 49, 69, 116). The increased risk of pneumonia is attributed to a general immunosuppression caused by an activation of the sympathetic nervous system and the hypothalamus‐pituitary‐adrenal gland axis causing lymphocyte apoptosis and dysfunction as well as parasympathetic, cholinergic pathways inhibiting innate immune cells occurring after central nervous system (CNS) injury and notably stroke (11, 33, 89). Another integral part of stroke pathology is the local inflammation in the affected brain area itself. It is well established that this local immune reaction contributes to ischemic pathology, influencing the death/survival of neurons and contributing to the functional outcome (15, 51). Besides the acute ischemic damage, research in recent years also showed that a chronic adaptive immune response in the ischemic hemisphere might contribute to the occurrence of poststroke dementia, which is another common complication after stroke and affects up to 30% of stroke survivors (28, 29, 86). The peripheral immune reaction dominated by stroke‐associated immunosuppression and infections and the central inflammatory reaction are closely linked. Stroke‐associated immunosuppression is thought to be a protective mechanism to limit brain inflammation, and thus, further damage (94). On the contrary, the increased occurrence of infections has been shown to function as a booster of CNS inflammation, as patients with poststroke infections show an increased CNS autoreactivity in the blood compared to stroke patients without infections, correlating with a worse clinical outcome (7, 8).

In this review, we will focus on the local CNS inflammation associated with stroke with a special focus on resident and infiltrating myeloid cells as well as their interaction with adaptive lymphocytes. Most of the data presented comes from animal models of cerebral ischemia, which only partly have been confirmed in human stroke patients so far. For the sake of clarity, the most important phenomena are summarized in Table 1, highlighting evidence from animal models and human data.

**Table 1:** Contribution of myeloid cells to brain inflammation after ischemia in animal models and human stroke.

Cell type	Animal studies	Human studies
Neutrophils	Early infiltration (or accumulation in perivascular spaces) within the first days after ischemia (34, 52) Release of proteases and reactive oxygen/nitrogen species (40, 100, 103) Phagocytosis, anti‐inflammatory N2 neutrophils (22, 41)	Infiltration within the first week after stroke (18, 70), but no significant infiltration in another study (121) MMP‐9 production causing blood–brain barrier breakdown and hemorrhagic transformation (46, 65, 101) Neutrophil extracellular traps contribute to thrombus formation and failure of recanalization (30, 85)
Microglia	Activation and change of morphology within hours after ischemia (75, 81) Loss in the ischemic core and accumulation in the penumbra (75, 81) Production of reactive oxygen/nitrogen species, inflammatory cytokines, and chemokines (9, 51) Phagocytosis, production of anti‐inflammatory cytokines, and growth factors (9, 51)	Loss in the ischemic core and proliferation in the penumbra within the first week after stroke (18, 70, 121) Expression of pro‐inflammatory markers CD68, p22phox, CD86, CD163, and iNOS during resorption stage (121) Production of TNFα (31) Decreased expression of the homeostatic marker P2RY12 in acute lesions, the penumbra, and resorption stage (121) Increased expression of the anti‐inflammatory marker CD206 in resorption phase (121) Phagocytosis of tissue debris (121)
Monocytes/macrophages	Infiltration of CCR2^+^ inflammatory monocytes peaks around day 3 after ischemia (39) Differentiation into CX3CR1^+^ macrophages (39) CCR2^+^ monocytes mainly accumulate in lesion core surrounded by CX3CR1^+^ macrophages/monocytes (39) Production of reactive oxygen/nitrogen species, pro‐inflammatory cytokines, and chemokines (51, 108) Phagocytosis, promotion of vascular stability, and angiogenesis (43, 83)	Difficulties in distinguishing resident microglia from infiltrating monocytes/ macrophages Substantial increase (especially in white matter lesions) of Iba1^+^TMEM119^−^ cells suggests recruitment of peripheral monocytes to ischemic lesion (121) Preferential accumulation around blood vessels in early lesions (121)

Table 1 summarizes key features of myeloid cell function after stroke, comparing data from animal models to results from human studies.

## Microglia and Infiltrating Myeloid Cells in Cerebral Ischemia

As CNS‐resident cells, microglia are the first immune cells to respond to acute ischemic damage in the brain. Microglia morphology is a good indicator of the cell’s activation status. Under homeostatic conditions, microglia have long and ramified processes. Upon activation, ramification is decreased and the cells adopt an amoeboid appearance. These different microglia morphologies are also observed in the ischemic hemisphere: in the lesion core, the number and length of microglia processes are significantly reduced as early as 8 hours after stroke. In the periphery of the lesion, microglia still show a ramified morphology, although with shorter processes than in the resting state (75). The majority of microglia accumulate in the periphery of the ischemic lesion, whereas the infarct core is mainly occupied by infiltrating myeloid cells in the first days poststroke. This activation of microglia primarily aims at clearing cellular debris that accumulates through neuronal cell death upon ischemia (9, 75, 81). In fact, mice deficient in triggering receptor expressed on myeloid cells 2 (TREM2) show impaired phagocytosis, increased infarct size, and worse functional outcome after stroke (58). During resolution of the acute inflammation, microglia produce anti‐inflammatory cytokines (IL‐10, TGFβ), growth factors (e.g., IGF‐1), and remove cellular debris by phagocytosis thus promoting angiogenesis and tissue reorganization (51). Following the same line of evidence, depletion of microglia in mouse models of cerebral ischemia has been associated with increased infarct sizes, aggravated neurological deficits, and enhanced brain inflammation accompanied by a stronger infiltration of leukocytes (neutrophils, T cells, natural killer cells, and monocytes/macrophages) (55). This effect was found to be mediated by a cross talk between microglia and astrocytes, as microglia depletion dramatically increased the production of pro‐inflammatory cytokines by astrocytes after stroke which directly induce neuronal apoptosis (55). *In vitro* and *in vivo* studies demonstrate that microglia can phagocytose infiltrating neutrophils, hence, reducing their cell number and contribution to the ischemic injury (25, 78, 81). Furthermore, microglia depletion alters neuronal function in the hours after ischemia. *In vivo* calcium imaging revealed that loss of microglia leads to the rapid development of neuronal calcium oscillations shortly after the induction of ischemia (105). Interestingly, the neurons showing these oscillations mostly died within 24 hours, and infarct sizes were increased when compared to mice without microglia depletion (105). The data presented so far highlights anti‐inflammatory effects of microglia in the early phase after stroke. Nevertheless, microglia also produce large amounts of pro‐inflammatory cytokines, reactive oxygen species, and other pro‐inflammatory mediators, thus, contributing to poststroke inflammation (51).

Activation of microglia after stroke is controlled by two mechanisms: 1) inhibitory interactions with neurons or other CNS cells are lost as well as 2) release of damage‐associated molecules upon ischemia. In the healthy brain, the interaction between neurons and microglia promotes a homeostatic microglia phenotype. For instance, the interaction between CD200 on neurons and its receptor, CD200R1 on microglia helps to keep microglia in a resting state. CD200 expression is known to decrease during tissue damage and aging, causing a reduced restraint on microglia activation (82). This could also occur during stroke when neuronal cells are lost because of the ischemic damage. In fact, CD200 knockout mice present a stronger neuroinflammatory response after stroke with increased immune cell infiltration and microgliosis. This was associated with a worsened functional outcome at 7 days after stroke (93).

Another important signal regulating microglia activation is provided by the fractalkine receptor CX3CR1 also expressed on microglia and its ligand CX3CL1 that is primarily produced by neurons. This is also an inhibitory signal, which is often lost in pathological conditions as neuronal expression of CX3CL1 is decreased or cells are lost. However, both detrimental and beneficial effects have been attributed to CX3CR1/CX3CL1 signaling in stroke. CX3CR1 or CX3CL1 knockout mice developed smaller lesions 1 day after cerebral ischemia. On the contrary, exogenous administration of CX3CL1 has also been shown to be protective during stroke in mice (82). It is hypothesized that depending on the context (ie, homeostatic brain or inflamed CNS containing infiltrating myeloid cells possibly also expressing CX3CR1), the CX3CR1‐CX3CL1 axis could have different effects on microglia/macrophage activation and brain inflammation (12).

Upon ischemia, neuronal function is greatly disturbed in the affected area. Stressed and dying cells start releasing danger molecules that directly act on microglia. Prominent molecules belonging to this category of danger‐associated signals are the nucleotides adenosine and uridine triphosphate (ATP, UTP), heat‐shock proteins (HSPs), and high mobility group box 1 (HMGB1). The released ATP acts on microglial P2X7 receptors and induces the release of pro‐inflammatory factors. Nucleotides also control microglia migration and phagocytosis (45), which can promote engulfment of still viable neurons, and therefore, contribute to brain damage upon stroke (9). On the contrary, damage‐associated molecules also bind to toll‐like receptors (TLRs) and scavenger receptors, equally inducing a pro‐inflammatory phenotype in microglia. Upon this activation, microglia rapidly release cytokines (IL‐1β, tumor necrosis factor α (TNFα), IL‐6), chemokines (CCL2, CCL3, CXCL2/3, CXCL8), and reactive oxygen species (51).

Following the activation of microglia, neutrophils are the first blood‐borne cells found in the ischemic hemisphere after stroke. In fact, neutrophil adhesion to the activated endothelium and rolling can be observed as early as 1 hour after experimental stroke (52). Whether the adhering neutrophils actually infiltrate into the ischemic hemisphere or remain trapped in perivascular spaces is controversial (34). Irrespective of this controversy, neutrophils have been shown to release neurotoxic substances such as reactive oxygen and nitrogen species, matrix metalloproteases (i.e., MMP9), and neutrophil elastase and contribute to blood–brain barrier disruption and ischemic damage (40, 100, 103). In fact, a higher neutrophil‐to‐lymphocyte ratio indicating increased numbers of neutrophils is associated with worse outcome and hemorrhagic transformation in human stroke patients (46, 65, 101). In addition, neutrophils release neutrophil extracellular traps consisting of DNA, histones, and granule proteins that contribute to thrombus formation and resistance to recanalization by thrombolysis (30, 62, 85). On the contrary, neutrophils are nowadays also considered to be heterogeneous cells and so‐called N2 or anti‐inflammatory neutrophils have also been shown to exert protective effects during cerebral ischemia (22, 41).

With regard to infiltrating monocytes, experimental studies also provide evidence for both detrimental and protective roles of invading monocytes/macrophages in brain ischemia. CCR2^+^Ly6C^hi^ monocytes are the main population of infiltrating monocytes in mouse studies of stroke (39, 72). Once in the ischemic hemisphere, they spread within the ischemic core and penumbra and differentiate into CX3CR1^+^ macrophages that can remain in the brain for extended periods of time (39). This spreading and differentiation is most likely controlled by chemokines and cytokines produced by local brain cells. Early after ischemia (i.e., 3 days), CCR2^+^ cells are evenly distributed in the damaged tissue. However, at 14 and 28 days after stroke, CCR2^+^ cells are mainly found in the lesion core, which is surrounded by CX3CR1^+^ monocytes/macrophages as shown by the transfer of CX3CR1^GFP/+^CCR2^RFP/+^ bone marrow into wild‐type mice. The number of CCR2^+^ monocytes peaks around day 3 post‐ischemia, whereas CX3CR1^+^ cells reach their peak much later, around 14–28 days after induction of ischemia (39).

The functional consequence of this monocyte/macrophage infiltration is controversial as different lines of evidence suggest protective as well as deleterious features. It was shown that selective deficiency of CCR2 in monocytes leads to a reduced infiltration after experimental stroke. This was associated with a worse functional outcome, a decrease of angiogenesis‐associated genes, and a reduced vessel density in the ischemic brain region (83). Furthermore, prevention of monocyte infiltration by clodronate‐liposomes and diphtheria toxin‐mediated monocyte depletion in CD11b‐DTR (diphtheria toxin receptor) mice increased the risk of hemorrhagic transformation of the infarct in the first week after ischemia, further highlighting the importance of monocytes/macrophages in angiogenesis and vessel integrity after stroke (43). In an additional study, circulating CCR2^+^ monocytes were depleted with an anti‐CCR2 monoclonal antibody during the first week after stroke, which caused a reduced recovery of motor function as late as 11 weeks post‐ischemia (113). However, deficiency of CCR2 or its ligand CCL2, and thus, reduced monocyte infiltration after stroke has also been associated with smaller infarct sizes, a reduction in pro‐inflammatory cytokine expression, and thus, a better functional outcome (27, 50). Additional studies could not find any effect of infiltrating monocytes on the outcome after ischemia. However, these studies did not assess cytokine production, angiogenesis, or vessel integrity (72, 96).

In addition to microglia and infiltrating monocytes/macrophages, the CNS harbors also resident macrophage populations called border‐associated macrophages (BAMs) that reside around blood vessels, in leptomeningeal spaces and the choroid plexus. In a rat model of ischemic stroke, it was shown that these cells rapidly respond to ischemic damage and contribute to the recruitment of granulocytes, increase vascular permeability, and participate in neurological dysfunction. This contribution of BAMs to the ischemic injury was studied in the very early phase of stroke (16 hours after induction) where peripheral immune cell infiltration is still negligible (84).

## Microglia–T Cell Interactions

Several experimental studies have shown that T lymphocytes modify stroke outcome, although the chronological sequence and types of T cell‐mediated effects have been controversial. Certain animal studies of stroke report T cell infiltration as early as 24 hours after ischemia, whereas in other cases, significant T cell infiltration is only observed in the chronic inflammatory phase from day 7 post‐ischemia onward (42, 104). Irrespective of the exact timing, T lymphocytes need signals to be attracted to the brain. Microglia may provide these signals through direct release of pro‐inflammatory molecules or by the chemoattraction of peripheral myeloid cells, which then in turn secrete the T cell‐attracting molecules.

The rapid release of TNFα, IL‐6, IL‐1β, and interferon γ (IFNγ) by microglia upon ischemia induces the upregulation of adhesion molecules such P‐Selectin, E‐Selectin, intercellular adhesion molecule (ICAM), and vascular cell adhesion molecule (VCAM) by endothelial cells (111). This prepares the ground for T cell adhesion and successful transmigration into the CNS. Through the release of chemokines such as CXCL2, CCL2, CXCL8, CXCL9, and CXCL10, microglia can directly attract T cells that express the corresponding receptors (51, 111).

It is now well established that T lymphocytes can polarize into different effector cells. T helper cells type 1 (Th1) and type 17 (Th17) promote neuroinflammation, whereas T helper cells type 2 (Th2) and regulatory T cells (Treg) rather dampen the inflammatory response in the brain (3, 4, 38, 64, 92). The generation of Th1 and Th17 cells requires certain cytokine profiles (IL‐12, IFNγ for Th1 and IL‐6, TGFβ, IL‐23 for Th17 cells), which can be provided by activated microglia and/or infiltrating myeloid cells (51, 67, 110). Once in the brain, the effector functions of T lymphocytes will be influenced by the local cytokine microenvironment, which is partly controlled by activated myeloid cells.

As mentioned above, after the first acute inflammatory phase, microglia is considered to adopt a rather anti‐inflammatory phenotype. Increased phagocytosis as well as the production of anti‐inflammatory cytokines such as IL‐4, IL‐10, and TGFβ generate a cytokine milieu favoring the generation of regulatory T cells that inhibit inflammation. In fact, Tregs infiltrate into the ischemic hemisphere and accumulate for as long as 30 days poststroke. Treg depletion markedly increases infarct volumes after middle cerebral artery occlusion (MCAO). Interestingly, depletion of Tregs also resulted in an increase of microglia cell numbers at day 3 after MCAO, most of which produced TNFα, highlighting the bidirectional communication between microglia and T cells (64). However, another study investigating depletion of Tregs in experimental stroke did not find any effects on functional outcome or immune cell numbers (104). The differences between these two studies might be in part explained by the use of different stroke models (transient vs. permanent ischemia) and the timing of Treg depletion (3 and 14 days post‐MCAO vs. 2 days before MCAO) (63).

On the contrary, infiltrating T cells produce cytokines that influence microglia function. Th1 cells are major producers of IFNγ, whereas Th17 cells produce mainly IL‐17. Microglia express receptors for both cytokines and can thus be influenced by their presence. Treatment of primary mouse microglia cultures with IFNγ induces the expression of IL‐1β, IL‐6, and TNFα (112). Coculture of primary human microglia with activated T cells also induces the expression of the pro‐inflammatory cytokines TNFα and IL‐12 (14).

Beyond CD4^+^ helper T cells, CD4^−^CD8^−^ γδ T cells were shown to infiltrate as early as 12–24 hours after ischemia and contribute significantly to tissue damage (42, 71). Infiltrating γδ T cells are major producers of the neurotoxic cytokine IL‐17. *In vitro* studies showed that TLR‐stimulated primary microglia are able to induce IL‐17 production in γδ T cells which in turn cause neuronal apoptosis (26). As TLR ligands, such as HSPs, HMGB1, and ATP, are abundantly present in the ischemic brain (73), similar mechanisms could take place *in vivo* after stroke. Furthermore, IL‐23 produced in the ischemic brain by microglia and macrophages further amplifies IL‐17 production by infiltrating γδ T cells (51, 99). Coculture of primary microglia with double‐negative T cells isolated from mouse spleens 3 days after MCAO was shown to increase the expression of the pro‐inflammatory molecules CD86 and CD16 while reducing the expression of the anti‐inflammatory marker CD206 in microglia (71).

The abovementioned studies mostly focus on the early phase after ischemia, meaning the first hours to 1 week post‐surgery. However, in recent years it has become increasingly obvious that stroke also generates long‐lasting, chronic immune responses in the affected brain regions (29, 98, 104, 118). Myeloid cells and relatively small numbers of pro‐inflammatory Th1 and anti‐inflammatory Treg cells mainly dominate the inflammatory response in the first week after stroke. However, stroke also generates antigen‐specific CD4 T cell responses (54). This delayed process involves antigen‐presenting cells, which phagocytose CNS proteins and present epitopes of them to CD4 T lymphocytes in the context of major histocompatibility complex (MHC) class II surface proteins. As activated microglia upregulate MHC class II, these cells could be in a unique position to prime or reactive infiltrating autoreactive T lymphocytes. In addition, microglia also express co‐stimulatory molecules such as CD80 and CD86 that are essential for a successful antigen‐dependent T cell activation (119). Antigen‐specific T cells occur with an increased frequency in the blood of stroke patients and have also been shown to infiltrate the ischemic brain in rodent models (60, 94). Through the transfer of myelin oligodendrocyte protein (MOG)_35‐55_‐specific T cells into Rag2^−/−^ mice, it was shown that these cells are reactivated in the ischemic brain and proliferate. In addition, cells with other specificities (i.e., to the peptides MOG_91‐108_ and MOG_103‐125_) were also locally expanded. This reactivation and clonal expansion was at least in part controlled by antigen presentation by microglia, as depletion of microglia reduced this phenomenon. Furthermore, these antigen‐specific T cells clearly play a deleterious role, as transfer of WT or ovalbumin‐specific T cells into Rag2‐/‐ mice generated smaller ischemic lesions compared to the transfer of brain antigen‐specific T cells (53).

Nevertheless, it is not definitely clear whether microglia play a similar role in WT mice in the presence of a polyclonal T cell pool. In the case of experimental autoimmune encephalitis (EAE), a rodent model of multiple sclerosis, it was shown that microglia, although the most abundant myeloid cells in the CNS, are not the main antigen‐presenting cells (APC) presenting myelin antigens to autoreactive T cells (44). Furthermore, several studies showed that CNS‐antigens are found in draining lymph nodes after stroke in patients and rodent models (88, 122). Therefore, it is possible that T cell activation through antigen presentation occurs in the periphery, before the autoreactive T cells infiltrate into the ischemic brain. Furthermore, dendritic cells are also observed in the ischemic hemisphere in rodent models and could present CNS antigens to infiltrating T cells (37).

Antigen presentation by microglia has also been shown to play a role in the induction of regulatory T cells. *In vitro* stimulation of microglia cultures with high‐dose IFNγ and MOG peptide was shown to increase proliferation of MOG‐specific effector T cells, whereas low‐dose IFNγ and MOG peptide preferentially led to the *de novo* generation and proliferation of MOG‐specific regulatory T cells. These microglia‐induced regulatory T cells significantly reduced disease severity when transferred to mice suffering from EAE. As speculated by the authors, this could present a mechanism for limiting inflammation and autoreactive responses after a first pro‐inflammatory phase likely also occurring in ischemic brain injury (32).

Whether antigen presentation occurs in the ischemic CNS and which myeloid cell type contributes to this phenomenon remains to be further studied.

## Microglia and B Lymphocytes

The role of B lymphocytes in cerebral ischemia has been neglected for a long time. First experiments carried out in µMT^−/−^ mice, which lack mature B cells revealed larger infarct volumes in the absence of B cells. The lack of B cells was accompanied by increased numbers of neutrophils, T cells, microglia, and infiltrating myeloid cells in the ischemic hemisphere (91). This effect is probably because of the release of the anti‐inflammatory cytokine IL‐10 by early infiltrating B cells, as adoptive transfer of IL‐10 producing B cells led to a reduction in infarct sizes, whereas IL‐10‐deficient B cells did not have an effect on acute infarct size (16, 91). However, other studies found no significant B cell infiltration into the ischemic brain or at least no clinical relevance for the infiltrating cells in the first week after stroke (42, 97).

RAG^−/−^ mice, lacking mature B and T cells were shown to have smaller infarcts compared to WT mice. Transfer of T cells abolished this phenomenon, whereas B cell transfer did not have this effect. This could either support the idea that B cell infiltration does not play a critical role early after ischemia or that a cross talk between B and T cells is needed to exert their effect on stroke‐associated inflammation (61).

The studies mentioned so far only analyzed the effect of B cells in the acute and subacute phase of experimental stroke. Here, B cells function mainly as cytokine producers. However, recent studies revealed that B lymphocytes might play an important role in the chronic phase occurring weeks after the onset of ischemia. Experimental studies demonstrated that B cells infiltrate in a delayed manner into the ischemic mouse brain and contribute to the occurrence of poststroke cognitive decline. This is thought to be mediated by the production of brain‐reactive antibodies that interfere with neuronal function (28, 29), as has been shown to occur in human stroke patients (23, 57, 79, 114). The occurrence of local, intrathecal antibody production after stroke has been observed for a long time (90, 107). B lymphocytes are present in the brains of stroke patients, and their numbers are significantly higher in those with poststroke dementia (28, 29).

Interestingly, the abovementioned studies as well as our own results show that the infiltrating B cells are not randomly spread but cluster within the ischemic lesion (10, 29). This organization is reminiscent of ectopic lymphoid structures (ELS) that occur in different chronic inflammatory and autoimmune diseases. In these disease settings, B cells differentiate into antibody‐secreting and memory cells in these ELS and contribute to the local clearance of pathogens or the propagation of autoimmune inflammation (2, 21, 87). In murine stroke models, CD138^+^ plasmablasts or plasma cells can be readily detected in the ischemic hemisphere (10, 29). Little is known about how B lymphocytes are attracted to the ischemic brain and how this organization is controlled. The formation of ELS closely mirrors the development of secondary lymphoid organs during which the expression of lymphoid chemokines (CXCL12, CXCL13, CCL19, and CCL21) by stromal cells, so‐called lymphoid tissue organizer cells, control the positioning of lymphocytes within the forming follicles in spleen and lymph nodes. Expression of these chemokines also occurs in ELS. However, in the adult organism during the formation of ELS, these chemokines are rather expressed by activated myeloid cells or lymphocytes. In the case of stroke, the situation is less clear. CXCL12 and its receptor CXCR4 are expressed constitutively in the brain but are upregulated after ischemia. In animal models, CXCL12 expression after ischemia is mainly attributed to endothelial cells and astrocytes and, besides regulating leukocyte chemotaxis, also contributes to angiogenesis and recruitment of neuronal progenitor cells. However, the main B cell‐attracting chemokine is CXCL13 (2, 20). It has been shown that *Cxcl13* mRNA is upregulated as early as 1 day after stroke and that protein production mainly occurs in the ischemic hemisphere by endothelial cells (74). However, this early after stroke, endothelial‐derived CXCL13 may not be responsible for the delayed infiltration of ELS‐forming B cells. A different experimental study analyzing the transcriptome of the ischemic penumbra found that *Cxcl13* mRNA is still upregulated as late as 8 weeks after stroke. In addition, such a delayed upregulation was also observed for *Cxcl12* and *Ccl19* mRNAs (17). Nevertheless, this study did not determine which cell types produce these chemokines or how their expression is regulated. Research on other inflammatory conditions of the CNS shows that microglia could potentially contribute to B cell infiltration through CXCL13 production. *Cxcl13* transcripts are increasing with age in retinal microglia (66). In addition, microglia have also been shown to produce CXCL13 during viral encephalitis (35).

Several reports show that CXCL13 can also be expressed by monocytes/macrophages in autoimmune diseases that are accompanied by the generation of ELS. This occurs for instance in the joints of rheumatoid arthritis patients and in the salivary glands of patients with Sjögren’s Syndrome (2, 6, 13, 20). In addition, stimulation with TNFα, IL‐10, or TLR7 ligands of human monocytes induces the secretion of CXCL13 (19). In the EAE mouse model, *Cxcl13* expression was shown to increase during the disease progression and was detected in CD11c^+^ dendritic cells in the spinal cord (5). Furthermore, a population of murine peritoneal macrophages constitutively expresses CXCL13 (1). Whether infiltrating monocytes/macrophages or dendritic cells also express CXCL13 and contribute to B cell infiltration in stroke remains to be determined.

Other studies show that B lymphocytes also infiltrate in remote areas that are not directly affected by the ischemic damage. Here, B cells have been shown to promote motor recovery and neurogenesis (80). The exact mechanism of this phenomenon is not clear yet. However, as the antibodies produced by infiltrating B cells likely bind CNS structures, it is possible that they also enhance phagocytosis through opsonization of cell debris, and thus, promote wound healing in the ischemic core. A similar phenomenon has been described in a study of peripheral nerve injuries. Here, natural antibodies accumulate in the damaged nerve tissue and promote clearance of myelin debris (109). As myelin and other cellular debris are also abundant in the ischemic brain, similar mechanisms could take place here, where the secreted antibodies could potentially further help microglia and macrophages during phagocytosis of damaged tissue components.

## Infection and Brain Inflammation Poststroke

As mentioned above, stroke is associated with an increased risk of infection. Studies in stroke patients have shown that notably the occurrence of pneumonia can increase autoimmune responses in the blood, and thus, also influence stroke outcome (8). Besides this effect on the peripheral, adaptive immune response, infections can also have an important impact on the innate immune response after stroke. It is well established now that peripheral infection can lead to activation and alteration of microglia phenotypes. Many different studies in animals showed that peripheral administration of LPS or killed/live bacteria can induce microglia activation as measured by increased expression of Iba‐1, MHCII, and pro‐inflammatory cytokines TNFα, IL‐1β, and IL‐6. Furthermore, similar experiments also showed an increased permeability of the blood–brain barrier after peripheral challenge with LPS or bacteria. Studies in sepsis patients revealed that systemic infection leads to a pro‐inflammatory activation of microglia shown by increased expression of CD68, iNOS, HLA‐DR, and CD86 on histological sections (120). Similar mechanisms could play a role during stroke and stroke‐associated infections. The peripheral immune challenge could further activate microglia, thereby increasing the inflammatory reaction, and fostering autoreactive immune responses in the brain after stroke.

In addition, it was shown that exposure to bacterial lipopolysaccharide (LPS) before the induction of ischemia can train microglia to produce higher amounts of IL‐1β. Microglia activation and neuronal damage was also increased in this model 7 days after stroke (115). Similar training effects are also observed in peripheral myeloid cells (47, 76, 77). Conversely, repeated LPS injections before the induction of stroke resulted in tolerized microglia with reduced expression of IL‐1β and reduced neuronal damage 7 days after ischemia (115). Therefore, a short‐term peripheral inflammatory stimulus such as stroke‐associated pneumonia could boost the immune reaction in the brain, whereas chronic stimulation likely leads to tolerance induction.

## Conclusion

**Figure 1:**
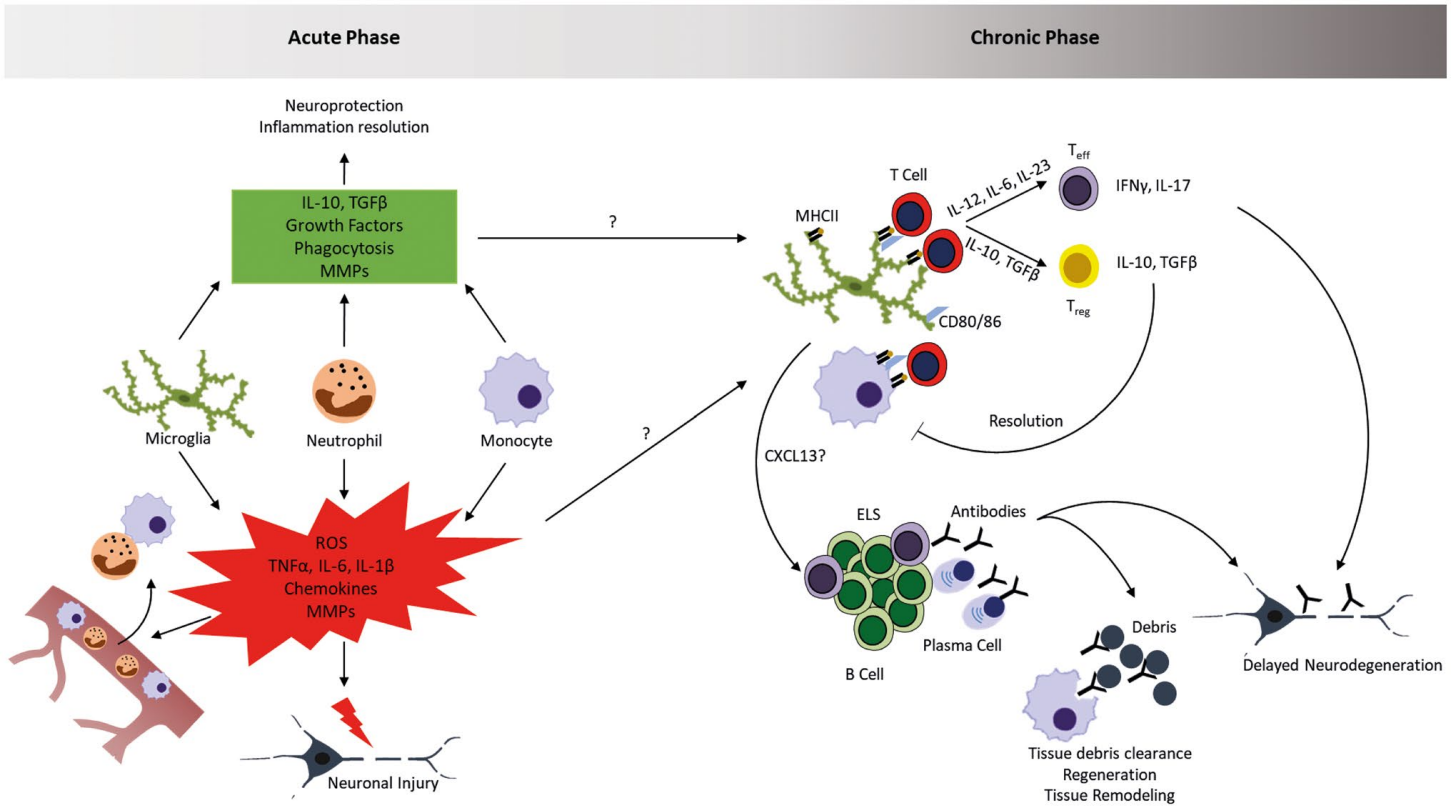
Inflammation in acute and chronic stroke lesions. Upon cerebral ischemia, tissue damage triggers the activation of microglia and the early infiltration of peripheral myeloid cells (neutrophils, monocytes). These cells contribute to tissue damage by the production of inflammatory mediators, reactive oxygen species (ROS) and MMPs, but also dampen this reaction and influence regeneration by releasing anti‐inflammatory mediators, growth factors, remove tissue debris and contribute to tissue remodeling. The mediators and molecular mechanisms influencing the transition from acute to chronic phase are currently unknown and a matter of active research. In this chronic phase where lymphocytes start infiltrating, myeloid cells can influence their function through antigen‐presentation and cytokine production. This leads to the generation of pro‐inflammatory T effector cells and anti‐inflammatory regulatory T cells. Through the release of chemokines, myeloid cells can potentially also influence the generation of B cell‐rich ectopic lymphoid structures (ELS) in which antibodies are produced. The generation of different T cell subsets and antibodies can promote delayed neurodegeneration, but potentially also contributes to tissue debris clearance and regenerative processes.

Neuroinflammation is a crucial part of stroke pathology in the acute and the chronic phase of the disease. Activation of resident immune cells as well as infiltration of peripheral leukocytes affects infarct development, resolution of the acute inflammation, tissue reorganization as well as motor and cognitive functional outcome (Figure 1). In this process, myeloid cells play a crucial role not only as initiators and perpetuators of inflammation, but also in the resolution phase. Recent advances in single‐cell technologies have revealed the whole spectrum of resting and activated myeloid cells after stroke with different beneficial and detrimental functions in mice and humans (48, 56, 59, 68, 95, 102, 106). Especially the recently described disease‐associated microglia (DAM) seem to represent a phenotype that is commonly observed in different neurodegenerative/neuroinflammatory disorders such as Alzheimer’s disease, amyotrophic lateral sclerosis, multiple sclerosis, and their animal models as well as during aging. It is proposed that these cells arise through a sequential two‐step differentiation, resulting in microglia cells that upregulate genes involved in phagocytosis, lysosomal degradation, and lipid metabolism. Their proposed function is to remove dying cells and cell debris in order to contain and resolve damage (24, 59). Whether DAMs are also generated after stroke, how they influence the acute phase of stroke as well as the chronic adaptive immune response is unknown to date. Furthermore, diversity of infiltrating myeloid cells is likely just as big as that of resident microglia and many of their functions probably remain understudied in the field of stroke immunology. To date, it is clear that following stroke, a complex immune reaction occurs in the ischemic brain. This reaction is influenced by the cross talk between innate and adaptive, peripheral and CNS‐resident immune cells. Furthermore, systemic events such as infections can modulate the inflammatory response following stroke. As outlined above, the function of different immune cells can change during the course of the poststroke immune reaction. In order to identify suitable therapeutic strategies that block deleterious effects and enhance protective and pro‐regenerative functions of immune cells, a detailed, molecular characterization of the different cell populations as well as their spatiotemporal interaction after stroke remains an essential topic to be studied in the future.
